# Hepatic Bile Acid Reuptake in the Rat Depends on Bile Acid Conjugation but Not on Agonistic Properties towards FXR and TGR5

**DOI:** 10.3390/molecules25102371

**Published:** 2020-05-20

**Authors:** Samuel A. J. Trammell, Jens S. Svenningsen, Jens J. Holst, Matthew P. Gillum, Rune E. Kuhre

**Affiliations:** 1 NNF Center for Basic Metabolic Research, Nutrient and Metabolite Sensing Program, Faculty of Health and Medical Sciences, University of Copenhagen, 2200 Copenhagen, Denmark; svenningsen@sund.ku.dk (J.S.S.); gillum@sund.ku.dk (M.P.G.); 2 Department of Biomedical Sciences, Faculty of Health and Medical Sciences, University of Copenhagen, 2200 Copenhagen, Denmark; jjholst@sund.ku.dk; 3 NNF Center for Basic Metabolic Research, Integrative Metabolism and Environmental Influences Program, Faculty of Health and Medical Sciences, University of Copenhagen, 2200 Copenhagen, Denmark

**Keywords:** hepatic bile acid reuptake, bile acid spill over, bile acids in plasma, portal vein versus periphery, FXR and TGR5 receptors

## Abstract

Farnesoid X receptor (FXR) and Takeda G-protein coupled receptor 5 (TGR5) are the two known bile acid (BA) sensitive receptors and are expressed in the intestine and liver as well as in extra-enterohepatic tissues. The physiological effects of extra-enterohepatic FXR/TRG5 remain unclear. Further, the extent BAs escape liver reabsorption and how they interact with extra-enterohepatic FXR/TGR5 is understudied. We investigated if hepatic BA reuptake differed between BAs agonistic for FXR and TGR5 compared to non-agonists in the rat. Blood was collected from the portal vein and inferior caval vein from anesthetized rats before and 5, 20, 30, and 40 min post stimulation with sulfated cholecystokinin-8. Plasma concentrations of 20 different BAs were assessed by liquid chromatography coupled to mass spectrometry. Total portal vein BA AUC was 3–4 times greater than in the vena cava inferior (2.7 ± 0.6 vs. 0.7 ± 0.2 mM x min, *p* < 0.01, n = 8) with total unconjugated BAs being 2–3-fold higher than total conjugated BAs (AUC 8–10 higher *p* < 0.05 for both). However, in both cases, absolute ratios varied greatly among different BAs. The average hepatic reuptake of BAs agonistic for FXR/TGR5 was similar to non-agonists. However, as the sum of non-agonist BAs in vena portae was 2–3-fold higher than the sum agonist (*p* < 0.05), the peripheral BA pool was composed mostly of non-agonist BAs. We conclude that hepatic BA reuptake varies substantially by type and does not favor FXR/TGR5 BAs agonists.

## 1. Introduction

Bile acids (BAs) are amphipathic, steroidal fat emulsifiers secreted from the liver and released from the gallbladder into the intestinal lumen to facilitate lipid absorption (Figure S1). Bile acids exist in primary or secondary forms. Primary BAs are synthesized in the liver, whereas secondary BAs result from bacterial metabolism in the lower small intestine and colon. Furthermore, BAs can be conjugated with either taurine or glycine, which both carry a negative charge at physiologic pH, forming bile acid salts with increased solubility. In their salt form, BAs emulsify fat and form micelles, which help solubilizing, transporting, and absorbing the lipase products of dietary lipids. Intestinal BAs are efficiently reabsorbed and returned to the liver via the enterohepatic circulation. In the liver, BAs are taken up, reconjugated, rehydroxylated, and resecreted to the gut (in rats) or directed to the gallbladder (in mice and humans), for concentration and storage until secretion is warranted [1,2]. Concurrent with their role in fat emulsification, BAs exert endocrine-like signaling activity through interaction with the two BA-sensitive receptors, the farnesoid X receptor (FXR, a nuclear receptor) and the Takeda G-protein coupled receptor 5 (TGR5, a cell surface receptor) [3,4], which are both highly expressed in the intestine and liver (however, for TGR5 only in non-parenchymal liver cells). Here, they both act to regulate BA abundance [3,4]. Additionally, TGR5 may regulate glucose, lipid and energy-metabolism [5] by increasing intestinal absorption through stimulation of intestinal secretion and peristaltic movements [6] and by stimulating the secretion of appetite- and blood-glucose regulating hormones via activation of basolaterally located TGR5 receptors [7,8,9]. FXR signaling seems restricted to regulation of BA synthesis from cholesterol through control of hepatic genes, secretion of BAs into bile [10,11], and regulation of the expression and secretion of fibroblast growth factor FGF19 (FGF15 in rodents) [12].

FXR and TGR5 are also found in non-enterohepatic tissues. TGR5 expression is high in the brain, spinal cord, smooth muscle tissue [13,14,15,16,17] and adipocytes, while FXR expression is high in the spleen, white adipose tissue, pituitary, adrenal gland, the kidneys, and the vasculature [18,19]. The physiological function of TGR5 and FXR in tissues outside the gastrointestinal tract and liver is not well understood, and it remains elusive to what extent and under which circumstances these receptors are activated. Hepatic BA reuptake is incomplete, rendering BAs detectable in the systemic circulation both during fasting and postprandially [20,21]. In the gut lumen and in the draining gut veins, the portal vein, and the liver, total BA concentrations range from 0.1 to 10 mM, but in the periphery, total concentrations are within the one to two digit micro molar range [20,21]. However, total BA concentration does not necessarily reflect potential for activation since some BAs are more potent agonists (LCA > CDCA > DCA, including glycine and taurine-conjugated isoforms) than others (CA and UDCA, also including glycine and taurine-conjugated isoforms) with agonistic BAs having EC50s towards FXR/TGR5 between 1–10 µM [22,23,24,25,26]. Together, the role of BAs as signaling molecules outside of enterohepatic circulation is unclear. To further our understanding of BA circulation, we stimulated BA secretion by sulfated CCK-8 in anesthetized rats and assessed concentration and composition by liquid chromatography coupled mass spectrometry (LCMS) of 20 different BAs in plasma collected from the portal vein (representing the enterohepatic BA return) and from the inferior vena cava (representing BAs that escaped liver extraction) (Figure S1). A second, related aim of our study was to elucidate whether the BA composition in systemic venous blood reflects the composition of BAs that return from the gastrointestinal tract to the liver.

## 2. Results

### 2.1. Total, Total Conjugated, and Total Unconjugated BA Concentrations in the Portal Vein and Vena Cava

**Figure 1:**
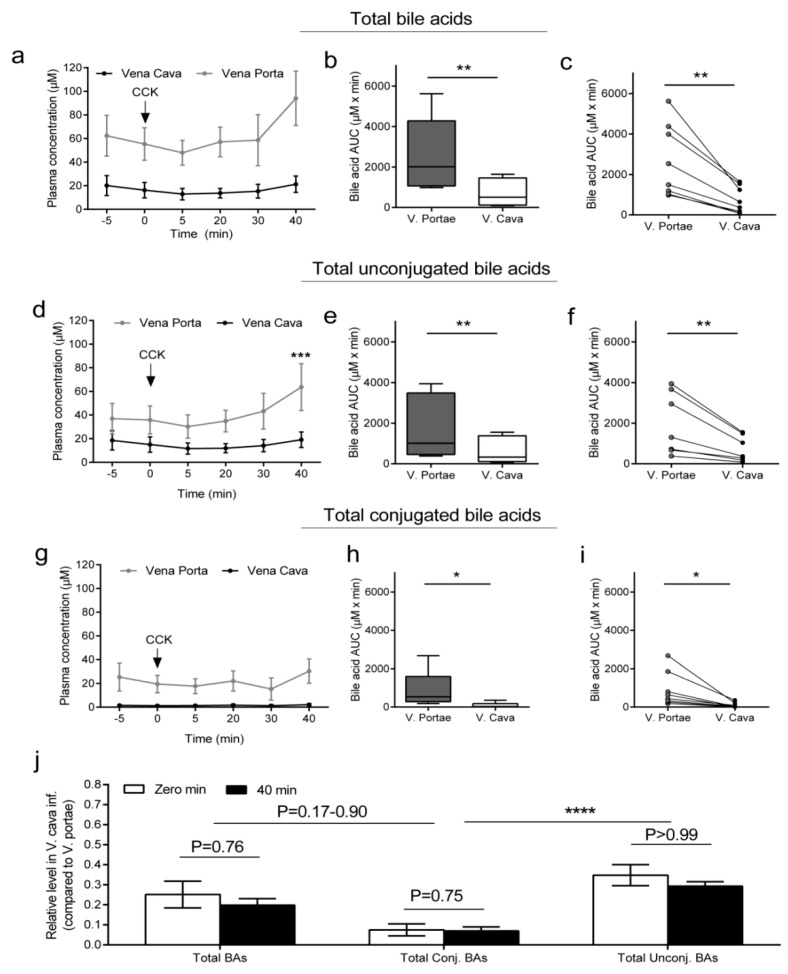
Total, total unconjugated, and total conjugated bile acid. Concentrations are shown at individual time points (means ± SEM, µM) and by AUC values from −5 to 40 min (µM × min) as well as by relative levels (means ± SEM) in vena cava (V. cava) compared to levels in vena portae (V. portae). AUC values are presented as box and whisker plots as well as connected individual AUC-values (µM × min). (**a**–**c**): Total bile acids (sum of total unconjugated and total unconjugated), (**d**–**f**): Total unconjugated bile acids, (**g**–**i**): Total conjugated bile acids, (**j**): Relative levels of total, total unconjugated, and total conjugated bile acid levels in V. cava compared to V. portae before injection of CCK (sulfated CCK-8) (zero point, white boxes) and 40 min after injection (black boxes). Zero point was calculated by taking the average of −5 and 0 min concentrations. Grey: Vena portae, black: vena cava. * *p* < 0.05, ** *p* < 0.01, **** *p* < 0.0001. Statistical significance was tested by paired student *t*-test. n = 8.

Total BA concentration at baseline was 58.9 ± 10.7 µM in vena portae (Figure 1a). As expected, the concentration in the vena portae was higher than in the vena cava inferior, where the plasma total BA concentration was 18.2 ± 5.18 µM (*p* < 0.001, Figure 1a). Overall, total BAs tended (*p* = 0.13) to increase around 40 min post CCK stimulation in vena portae, whereas the concentrations in vena cava inferior did not change (*p* = 0.47). Total BA AUC values in vena portae were 4 times higher than total BA AUC in vena cava inferior (*p* < 0.01, Figure 1a–c). Unconjugated BAs constituted most of the BA pool in both the portal vein and vena cava inferior. On average, basal unconjugated BAs were approximately twofold higher in the vena portae compared to the vena cava (36.4 ± 12.1 and 16.8 ± 7.30 µM in the vena portae and vena cava, respectively (*p* = 0.19, Figure 1a)). At 40 min, concentrations in vena portae were 63.7 ± 19.8 µM (*p* < 0.001, compared to 0 min) and 19.1 ± 6.56 µM in vena cava inferior (*p* = 0.70 compared to 0 min, Figure 1a). Total AUC values were approximately three times higher in vena portae than vena cava inferior (Figure 1d,e, *p* < 0.01). The concentrations of conjugated total BAs at 0 min were 22.5 ± 9.32 µM in vena portae and 1.43 ± 0.57 in vena cava inferior, but neither was significantly different at 40 min (*p* > 0.90). Total AUC for conjugated BAs was approximately 10-fold higher in vena portae than in vena cava inferior (*p* < 0.05, Figure 1h,i). The concentration ratios at 0 min and 40 min across the liver of total bile acids, as well as the total conjugated BAs and unconjugated BAs, did not differ, but the relative extraction (i.e., the amount presumed to be absorbed by the liver) of conjugated BAs was fivefold higher than the extraction of unconjugated BAs.

### 2.2. Concentrations of Primary BAs in the Portal Vein and Vena Cava

Cholic acid (CA) and tauro (T) cholic acid concentrations were threefold higher in vena portae compared to chenodeoxy cholic acid (CDCA) and TCDCA. CCK increased the concentration of CA in vena portae by approximately 50% (*p* < 0.001) but did not affect TCA, CDCA, or TCDCA concentrations (*p* = 0.13–0.99), which in case of CDCA and TCDCA remained low throughout the experiment (Figure 1 and Table 1). The fractional concentration of individual bile acids in vena cava inferior compared to respective concentrations in vena portae varied greatly among BAs and was at 0 min: TCA = 0.08 ± 0.03 µM, TCDCA = 0.19 ± 0.03 µM, CA = 0.30 ± 0.03 µM; CDCA = 0.61 ± 0.06 µM. Fractions did not differ significantly (*p* = 0.71–0.99) at time 40 min versus 0 min, except for CDCA which was mildly lower (40 min: 0.40 ± 0.03 µM, *p* < 0.01) (Figure 1 and Table 1). Concentrations of glycine-conjugated isoforms of the respective BAs were below quantification limit, except for glycine-CA which at 40 min was 4.63 ± 1.75 µM in vena portae and 0.97 ± 0.25 µM in vena cava inferior and did not change significantly throughout the experiment (data not shown).

### 2.3. Concentrations of Secondary BAs in the Portal Vein and Vena Cava

Concentrations of the secondary BAs DCA, TDCA, ursoDCA (UDCA), and TUDCA were generally low (<2.5 µM) in vena portae at 0 min, expect for UDCA which was 7.02 ± 3.28 µM (Table 1). In vena cava inferior, concentrations of DCA, UDCA, and TUDCA at 40 min were 1.5–2-fold higher than at 0 min (*p* < 0.05, compared to respective basal levels), (whereas TDCA concentrations remained near the quantification limit (Table 1). AUCs were ~3-fold lower for DCA in vena cava inferior than in vena portae (*p* < 0.05) and tended to be lower in vena cava inferior (3–5-fold) for TDCA (*p* = 0.11), UDCA (*p* = 0.08), and TUDCA (*p* = 0.11) (Table 1). Fractional individual BAs in vena cava inferior compared to respective concentrations in vena portae were at 0 min: DCA = 0.43 ± 0.03; UDCA = 0.25 ± 0.39; TDCA = 0.80 ± 0.09; TUDCA = 0.47 ± 0.13). Fractions for the individual BAs did not differ significantly between times 0 and 40 min (*p* = 0.23–0.99) (Table 1).

### 2.4. Concentrations of Murine Specific BAs in the Portal Vein and Vena Cava

Murine CA (MCA) alpha and MCA beta concentration in vena cava inferior was at time 0 min 1.95 ± 0.87 and 2.65 ± 0.94 µM, respectively. Taurine-conjugated forms of these were in vena cava inferior at same time point 2.01 ± 0.82 µM and 5.29 ± 2.60 µM, respectively. MCA alpha and TMCA beta concentrations did not change over the time course of the study, but MCA beta and TMCA alpha were 2–3-fold higher at time point 40 min (*p* < 0.01) (Table 1). Corresponding concentrations in vena cava inferior were at time point 0 min and 40 min: MCA alpha: 0 min: 0.93 ± 0.44 µM, 40 min = 1.00 ± 0.42 µM; MCH beta: 0 min = 1.23 ± 0.49 µM, 40 min = 1.85 ± 0.58 µM; TMCA alpha: 0 min = 0.29 ± 0.08 µM, 40 min = 0.36 ± 0.10 µM; TMCA beta: 0 min = 0.25 ± 0.07 µM, 40 min = 0.39 ± 0.14 µM, i.e., no significant differences between 0 min and 40 min. AUCs in vena portae were approximately 2-, 3-, 5-, and 8-fold higher in than in vena cava inferior for MCA alpha, MCA beta, TMCA alpha, and TMCA beta, respectively (*p* < 0.05, Table 1). Fractional concentration of individual bile acids in vena cava inferior compared to respective concentrations in vena portae was not significantly different at time 0 min and 40 min for the individual BAs (*p* = 0.70–0.88), but varied among BAs (Table 1). Glycine-conjugated forms of the respective BAs were below quantification limit.

### 2.5. Concentration of FXR and TGR5 Agonistic BAs in Peripheral Blood is below Activating Concentrations, and Hepatic Extraction of BAs is Independent of Their Potency and Efficacy towards These Receptors

**Figure 2:**
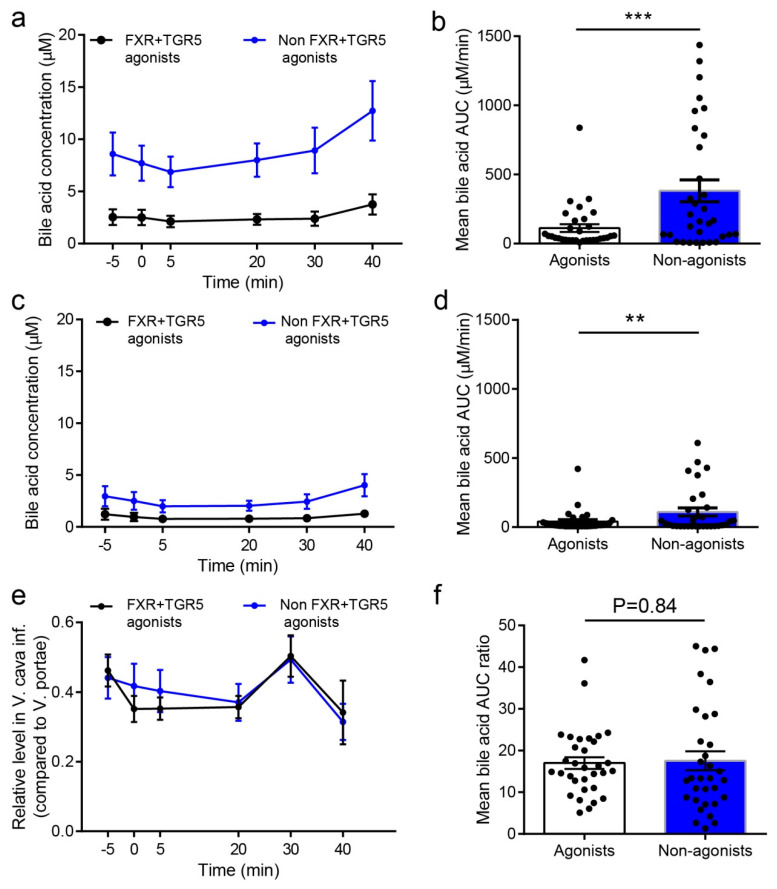
FXR and TRG5 activating bile acids and non-activating bile acids. Levels of bile acids that respectively are agonist and non-agonists for FXR and TGR5 are shown. Following BAs were, based on previous studies on FXR activation and TGR5 activation [22,23,24,25,26], categorized as FXR and TGR5 agonists: Deoxycholic acid, Chenodeoxycholic acid, Lithocholic acid (both unconjugated and glycine- and taurine-conjugated isoforms). Cholic acid and Ursodeoxycholic acid were classified as non-agonists based on same studies. Murine specific BAs were not included in the analysis because of lack of information with regards to agonistic/non-agonistic functions on FXR and TGR5. (**a**,**b**): Mean concentration of FXR/TGR5 agonists and non-agonists at individual time points (mean ± SEM, µM) (µM) in vena portae (a), vena cava inferior (c). (**c**,**d**): AUC values (−5 to 40 min) of FXR/TGR5 agonists and non-agonists (mean values ± SEM) in vena portae (b) and vena cava inferior (d). Individual values are indicated with dots. (**e**): Relative level (mean ± SEM) of FXR/TGR5 agonist and non-agonists (conc. vena cava inferior/conc. Vena portae). (**f**): Mean bile acid AUC ratio (−5 to 40 min) of FXR/TGR5 agonist and non-agonists (conc. vena cava inferior/conc. Vena portae) (**e**). Individual values are indicated with dots. FXR/TGR5 agonist was defined as chenodeoxycholic acid, taurodeoxycholic acid, deoxycholic acid, and taurodeoxycholic acid, whereas non-agonist was cholic acid, taurocholic acid, ursocholic acid, and tauroursocholic acid. Murine-specific BAs (MCA alpha, MCA beta, and taurine-conjugated forms of these) were not included in the analysis because of lack of information regarding FXR and TGR5 activation. Statistical significance was tested by paired student *t*-test, ** *p* < 0.01, *** *p* < 0.001, *n* = 8.

It is well established that the liver retains a large proportion of BAs from the portal vein [27]. We assessed whether FXR/TGR5 agonistic BAs were preferentially allowed to pass into peripheral circulation by comparing the concentrations and compositions in the vena portae to those in the vena cava. BA concentration in the vena cava varied substantially by type of BA. The apparent liver reabsorption fraction ranged from 10%–45% (Table 1). When stratified into agonists and non-agonist for FXR/TGR5, the hepatic extraction ratios were similar (Figure 2a,b and Table 1), suggesting that BA reuptake in the liver is independent of potency towards FXR/TGR5. However, comparing to observations in the vena portae (Table 1), the mean non-agonist BA concentrations were threefold higher in vena cava inferior (AUCs: conjugated BAs = 112 ± 28.3 µM/min, non-agonists = 382 ± 78.6 µM/min, *p* < 0.01. n = 8, Figure 2a,b and Table 1). The relative level of agonistic and non-agonistic BAs towards FXR and TGR5 in vena cava inferior compared to vena portae was similar at the individual time points and over the course of the experiment (mean AUC ratios) (Figure 2e,f, *p* > 0.05, n = 8).

## 3. Discussion

Compared to the gastrointestinal tract and liver, extra-enterohepatic FXR and TGR5 regulation has been studied to a limited extent with focus mainly on distribution of tissue expression, not on ligand concentration. TGR5 expression is high in the brain, spinal cord, smooth muscle tissue, and adipocytes [13,14,15,16,17], while FXR is expressed highly in the spleen, white adipose tissue, pituitary, adrenal gland, the kidneys, and the vasculature [18,19]. The extent of receptor activation by specific BA isoforms in these tissues and the down-stream effects of activation remain largely unknown. We hypothesized that if BAs act as agonists post liver, hepatic reuptake of potent FXR/TGR5 BAs agonists would differ from non-agonists. We investigated concentrations of 20 different BAs in rat plasma collected immediately before liver reuptake (vena portae) and after perfusion through liver (vena cava inferior).

We and others [28] have shown that human TGR5 is activated with EC_50_ values of 0.4–10 µM in the following hierarchy of potencies: lithocholic acid (LCA) > DCA > CDCA, independent of conjugation, whereas neither CA nor UDCA (both conjugated and unconjugated isoforms) activated the receptor at concentrations up to 10 µM [22,23]. Human FXR is differentially activated in the following order of potencies: CDCA > DCA > LCA with EC_50_ values in the range of 10–100 µM [24,25,26]. In the case of TGR5, CA and UDCA were poor agonists or did not activate the receptor [24,25,26]. In humans, fasting total plasma BA-concentrations collected from a peripheral vein are normally in the single-digit micromolar range and increase by a factor of 2–3 after intake of fat-rich meals [20,29,30]. However, total concentrations do not allow predictions regarding receptor activation, given ligand specificity. We found that total BA concentrations in vena portae were 4–5 times higher than in peripheral veins, which is in agreement with other studies in rats, pigs, and monkeys (based on quantification of total BAs) [31,32,33]. In humans, the relative hepatic BA reuptake seems to be even higher, as the total BA concentration in peripheral blood is only 10%–16% of the concentration in the portal vein [21,31,32]. Whether this difference results from inter-species difference with respect to the dilution of the splanchnic in to the systemic circulation (the splanchnic bed receives around 1/4 to 1/3 of the cardiac output) or results from more efficient hepatic reuptake in humans is not well characterized. We found that the concentration of conjugated BAs in vena cava inferior was as a whole less than 1/10 of the concentration in vena portae, whereas the ratio for unconjugated BAs was around 1/3. Since measurements were within animals over time, differences in the splanchnic bed–cardiac output ratio cannot have influenced the measured concentrations, and hepatic reuptake of conjugated BAs is therefore about three times more efficient than their unconjugated counterparts. The mechanisms underlying preferential re-uptake of conjugated BAs warrants further investigation but may relate to differing transport capacities of the primary hepatic transporters (Na^+^ taurocholate cotransporting protein) and non-conjugated BAs (organic anion transporting protein family) into the hepatocytes [34]. Furthermore, the variability of reuptake may also be influenced by differences in hydrophobicity with more hydrophobic BAs more easily diffusing through the plasma membranes, and ultimately into the liver. In the current study, no clear evidence was found to support this as hydrophobicity (measured by retention time on a Phenomenex PS C18) did not correlate with hepatic retention; neither in case of non-conjugated BAs (Pearson correlation = 0.609, *p*-value = 0.200) nor their corresponding conjugated forms (Pearson correlation = 0.109, *p*-value = 0.837). However, since our study was not designed and powered to address this aspect, future work with expanded sample sizes is needed to appropriately address this possible explanation. However, as activation of FXR/TGR5 differs among BAs and is unaffected by conjugation, total concentrations of conjugated and unconjugated BAs provides little information with regard to potential extra-enterohepatic FXR and TGR5 activation. We therefore quantified the concentrations of unconjugated and conjugated CA, CDCA, DCA, UDCA, MCA alpha, and MCA. In the portal plasma, CA and TCA predominated and composed approximately 40% of the total BA concentration, whereas CDCA and DCA and their taurine-conjugated counterparts each accounted for only 2%–7%. These ratios are in agreement with another study in rat [35]. In humans, CA is also the predominant BA in the portal vein, and in contrast to the rat, CDCA is the second-most abundant BA and makes up about half the concentration of CA [21]. This species difference may in part be attributed to Cyp2c70 expression in mice and rats but not humans. Cyp2c70 catalyzes hydroxylation of CDCA to form the murine specific BAs alpha-MCA [36]. Indeed, the portal concentration of MCA combined with the concentration of CDCA measured in our study is about half the total concentration of CA.

Of note, concentrations of different BA specimens in the periphery varied greatly and differed by almost 50-fold between the most (UDCA) and the least abundant (TDCA) BA. Moreover, the ratio difference between portal and peripheral concentrations for the quantified BAs varied widely, ranging from 0.07 µM (TCA) to 0.46 µM (MCA alpha). However, when grouped into agonist for FXR/TGR5 (CDCA, TCDCA, DCA, TDCA) and poor-agonists/non-agonists (CA, TCA, UDCA, TUCDA), average ratios between concentrations in vena portae and vena cava inferior were similar between groups, suggesting hepatic reuptake of BAs is regulated by hepatic BA delivery rather than ability to activate FXR/TGR5. As non-agonists accounted for more than 2/3 of the total BA pool in the portal vein, peripheral concentrations of non-agonist BAs dominated and the average concentration of agonist BAs amounted to around 1 µM at baseline and peaked at approximately 1.3 µM. As a reference, EC_50_ values for these agonists on FXR/TRG5 are 1–10 µM, suggesting BAs could not activate either receptor in this study.

A general observation in our study is that concentrations of BA as well as the transhepatic BA ratios differed significantly between rats. Although we were unable to narrow down the underlying reason for this pronounced animal-to-animal variation, other studies also showed great individual variation in monkeys and humans [21,31,33,37,38], suggesting that these figures are generally highly variable between individuals. The mechanisms underlying this inter-individual variability may include factors such as luminal BA appearance, gut microbiota composition, BA transporter genotypes, BA synthetic capacity, and gut motility [32]. Moreover, BA concentrations are regulated through a complex interaction between diet and circadian rhythm [39]. In this study, rats were sacrificed within a rather narrow time span (10 am and 1 pm) and although not measured, food intake is anticipated to be low in the hours leading up to the experimental period since the dark period ended at 6 am. Variations resulting from difference in circadian rhythm and feeding status between study days is therefore unlikely to have influenced our results to a major extent. Whether a different time of day would have led to similar or different results is an interesting and potentially important question that requires further investigation.

A weakness of our study is that the injected CCK only resulted in moderately increased BA secretion, and it remains to be investigated whether stronger stimuli (e.g., a fat-rich solution) would have resulted in different or similar hepatic reuptake of the investigated BAs and extra-enterohepatic plasma concentrations that were more within respective EC50s for FXR/TGR5 activation.

## 4. Materials and Methods

### 4.1. Animal Studies

#### 4.1.1. Ethical Considerations

Animal studies were conducted with permission from the Danish Animal Experiments Inspectorate (2013-15-2934-00833) and approved by the local ethical committee (Department of Experimental Medicine, University of Copenhagen). Animal studies were conducted in accordance with the EU Directive 2010/63/EU and guidelines of Danish legislation governing animal experimentation (1987) and the NIH (publication number 85-23) and were designed to minimize pain or discomfort to the animals.

#### 4.1.2. In Vivo Study

Male Wistar rats were obtained from Janvier (Saint Berthevin Cedex, France) and housed in pairs under standard conditions with ad libitum access to chow and water and a 12:12 h light and dark cycle. Rats were allowed to acclimatize for at least one week before the study. Studies were carried out between 10 am and 1 pm. Rats were allowed free access to chow and water in the hours leading up to the experiment, but as the dark period ended at 6 am, the rats can in our experience be considered to be semi-fasted at study initiation. Rats (mean weight ± standard error of the mean (SEM) = 492 ± 34 g) were anesthetized with a subcutaneous injection of Hypnorm/midazolam (0.079 mg fentanyl citrate + 2.5 mg fluanisone + 1.25 mg midazolam/mL: 0.3 mL/100 g body weight). The abdominal cavity was opened and a needle inserted into the inferior caval vein (vena cava inferior) concurrent with insertion of a non-obstructing plastic catheter into the portal vein. Blood (600 µL/time point) was withdrawn simultaneously from the two veins into EDTA coated syringes (EDTA: cat. no. 03690, Sigma Aldrich, Brøndby, Denmark). The samples were immediately transferred into 1.5 mL EDTA-coated tubes (cat. no. 200 K3E, Microvette; Sarstedt, Nümbrecht, Germany). To prevent clot formation and replace fluid lost from blood collection, the needle and catheter were both flushed with 400 µL isotonic saline (room temperature) immediately after sample collections. Samples were drawn at the time points −5, 0, 20, and 40 min and transferred onto ice and centrifuged (1650× *g*, 15 min, 4 °C) within half an hour of collection. Supernatant was transferred to fresh centrifuge tubes and immediately stored at −20 °C until analysis as described below. After collection of the zero min sample, sulfated CCK-8 (ammonium salt, cat. No. 4033010.0001, Bachem, Bubendorf, Schweiz) was injected at a dose of 20 nmol/kg body weight (~260–550 µL depending of body weight). CCK-8, diluted in isotonic saline supplemented with 10% (*w/v*) human serum albumin (cat. No. 12666, Emd Millipore Corp., Bedford, MA, USA) was injected intravenously through the needle inserted in the inferior caval vein to stimulate bile acid release into the intestine. In total ~6 mL blood was withdrawn from each animal, corresponding to ~18% of the theoretical total blood volume of 33.2 mL (estimated by: blood volume (mL) = 0.06 × body weight (g) + 0.77, as described previously [40]).

### 4.2. Biochemical Measurements

10 µL of plasma was extracted with 80 µL of 87.5% methanol containing 12 pmol of internal standard. Extracts were clarified by centrifugation (16,100× *g*, 10 min, 22 °C) and dried via speed vacuum. Quality controls were composed in triplicate at three different concentrations (0.25, 2.5, and 12.5 μM) in the presence of liquid chromatograph mass spectrometry grade water or human plasma. The quality controls as well as a standard curve were processed in the same manner as samples. A blank was composed in the presence and absence of human plasma. Quality controls were interspersed at even intervals throughout the queue. Samples were analyzed in a random order to control for systematic error. Metabolites were separated, detected, and quantified as described [41]. Briefly, except for chenodeoxycholic acid (CDCA), BA concentrations were calculated using a standard curve normalized to internal standards. CDCA was quantified by comparison of its area to the area of the exogenously added internal standard [42]. The limit of quantification was set as the lowest standard concentration value that could be distinguished from zero within the standard curve (0.03 µM). For CDCA, the lowest concentration on the standard curve for DCA was used (0.03 µM). When corrected for sample dilution the limit of quantification was 0.15 µM. Taurine- and glycine-conjugated (total conjugated), total non-conjugated, and total BA concentrations were calculated by summing the appropriate BA concentrations.

### 4.3. Data Analysis and Statistics

Statistical methods of this study were reviewed by Prof. Jens Juul Holst. Data are presented as mean concentrations ± SEM. Values below the limit of quantification were imputed using the lowest standard concentration. BAs whose concentrations were below the limit of quantification in >50% of samples were excluded from analyses. These BAs were glycochenodeoxycholic acid, glycodeoxycholic acid, glycoursodeoxycholic acid, lithocholic acid, muricholic acid gamma, taurolithocholic acid, and tauromuricholic acid gamma Graphs were constructed in GraphPad Prism 6 (La Jolla, CA, USA) and collected and prepared in Adobe Illustrator (San Jose, CA, USA). Test for statistical significance was performed in GraphPad Prism. Significance was assessed at individual time points against own baseline (average of −5 and 0 min samples, in vena cava inferior and vena portae, respectively) by two-way ANOVA for repeated measurements followed by Tukey’s multiple comparison test. Significance amongst relative level of BAs was tested by two-way ANOVA followed by Tukey’s multiple comparison test, whereas significance between respective AUC’s in v. portae and v. cava inferior was tested by paired *t*-test. *p* ≤ 0.05 was considered significant for all tests. Pearson correlations and significance of correlation were performed using GraphPad Prism 8 (La Jolla, CA, USA).

## 5. Conclusions

We find that total BA concentrations in the vena cava inferior were 4–5 times lower than the total concentration in vena portae. Conjugated BA hepatic reuptake was on average 3–4 times more efficient than the reuptake of unconjugated BAs, but reuptake varied greatly between individual BAs. Collectively, BAs that are agonist for FXR/TGR5 were reabsorbed by the liver to the same extent as non-agonists. The concentration of non-agonists was 2–3-fold higher in vena portae than agonists, suggesting regulation of extra-enterohepatic FXR/TGR5 activity is mediated by hepatic delivery of BAs rather than by specific/differential hepatic reuptake of agonistic BAs towards FXR/TGR5.

## Figures and Tables

**Table 1:** Bile acids in plasma from vena portae and vena cava inferior.

	Plasma from Vena Portae			Plasma from Vena Cava Inferior			
Bile acid	Baseline (µM)	40 min (µM)	AUC (µM x min)	Baseline (µM)	40 min (µM)	AUC (µM x min)	Ratio: V. Cava/V. Portae
*Collected*							
Total	58.9 ± 10.7	87.4 ± 25.1	2650 ± 634	18.3 ± 5.18	28.0 ± 8.68	726 ± 231 **	0.25 ± 0.07 (0 min) 0.20 ± 0.03 (40 min)
Total conjugated	22.5 ± 9.32	30.4 ± 10.1	899 ± 317	1.43 ± 0.57	2.18 ± 0.91	92.4 ± 45.1 *	0.08 ± 0.03 (0 min) 0.07 ± 0.02 (40 min)
Total unconjugated	36.4 ± 12.1	63.7 ± 19.8	1752 ± 535	16.8 ± 7.30	19.1 ± 6.56	636 ± 222 **	0.35 ± 0.05 (0 min) 0.29 ± 0.02 (40 min)
*Primary BAs*							
CA	13.6 ± 4.10	21.9 ± 5.87	190 ± 50.4	4.67 ± 1.73	5.54 ± 1.35	658 ± 168 **	0.30 ± 0.03 (0 min) 0.28 ± 0.02 (40 min)
TCA	10.5 ± 4.39	12.6 ± 4.38	404 ± 153	0.56 ± 0.19	0.81 ± 0.32	37.4 ± 17.2 *	0.08 ± 0.03 (0 min) 0.07 ± 0.02 (40 min)
CDCA	4.41 ± 2.67	6.50 ± 3.21	186 ± 99.4	2.89 ± 1.18	2.34 ± 1.01	93.3 ± 50.2	0.61 ± 0.06 (0 min) 0.43 ± 0.03 (40 min)
TCDCA	2.30 ± 0.91	3.34 ± 0.96	95.1 ± 25.8	0.31 ± 0.08	0.37 ± 0.09	16.0 ± 5.31 **	0.19 ± 0.03 (0 min) 0.13 ± 0.01 (40 min)
*Secondary BAs*							
DCA	2.45 ± 0.85	4.87 ± 1.66	128 ± 43.4	0.97 ± 0.35	1.14 ± 0.35	43.2 ± 12.9 *	0.43 ± 0.03 (0 min) 0.33 ± 0.08 (40 min)
TDCA	0.92 ± 0.18	1.34 ± 0.39	39.4 ± 10.5	0.18 ± 0.17	0.20 ± 0.035	8.86 ± 1.20	0.25 ± 0.04 (0 min) 0.19 ± 0.03 (40 min)
UDCA	7.02 ± 3.28	15.7 ± 7.60	387 ± 179	5.44 ± 2.89	6.43 ± 3.285	197 ± 91.3 *	0.25 ± 0.04 (0 min) 0.19 ± 0.03 (40 min)
TUDCA	1.58 ± 0.81	3.69 ± 1.80	387 ± 179	0.26 ± 0.07	0.40 ± 0.19	197 ± 91.3	0.47 ± 0.13 (0 min) 0.39 ± 0.13 (40 min)
*Murine specific BAs*							
MCA alpha	1.95 ± 0.87	4.23 ± 1.77	97.4 ± 39.4	0.93 ± 0.44	1.00 ± 0.42	34.8 ± 13.6 *	0.46 ± 0.04 (0 min) 0.37 ± 0.06 (40 min)
MCA beta	2.65 ± 0.94	6.42 ± 2.02	144 ± 46.3	1.26 ± 0.49	1.85 ± 0.58	60.0 ± 15.9 *	0.42 ± 0.04 (0 min) 0.69 ± 0.37 (40 min)
TMCA alpha	2.01 ± 0.82	2.98 ± 0.91	81.9 ± 25.7	0.29 ± 0.08	0.36 ± 0.10	14.9 ± 5.09	0.25 ± 0.05 (0 min) 0.16 ± 0.03 (40 min)
TMCA beta	5.29 ± 2.60	6.52 ± 2.22	203 ± 78.1	0.25 ± 0.07	0.39 ± 0.14	20.7 ± 10.9 **	0.16 ± 0.06 (0 min) 0.08 ± 0.01 (40 min)
*TGR5/FXR*							
Agonists	2.52 ± 0.74	3.75 ± 0.97	112 ± 28.3	1.09 ± 0.48	1.27 ± 0.33	40.4 ± 13.7 **	0.38 ± 0.04 (0 min) 0.25 ± 0.03 (40 min)
Non-agonists	8.73 ± 1.64	12.7 ± 2.85	382 ± 78.6	2.74 ± 0.91	4.03 ± 1.06	110 ± 29.4 ***	0.25 ± 0.03 (0 min) 0.31 ± 0.05 (40 min)

Data are shown as mean ± SEM; grey: vena portae, black: vena cava inferior. Concentrations (µM) at baseline (average of −5 and 0 min) and 40 min after injection of CCK-8 are shown. AUC (µM × min) values were calculated using all time points in the experiment (from −5 to 40 min). AUC ratios between vena cava (V. Cava) and vena portae (V. Portae) were calculated by normalizing individual concentrations of the respective bile acids in vena portae to the corresponding concentration in vena cava inferior. Following BAs were, based on previous studies on FXR activation and TGR5 activation [22,23,24,25,26] categorized as FXR and TGR5 agonists: Deoxycholic acid, Chenodeoxycholic acid, Lithocholic acid (both unconjugated and glycine- and taurine-conjugated isoforms). Cholic acid and Ursodeoxycholic acid were classified as non-agonists based on same studies. Murine specific BAs were not included in the analysis because of lack of information with regards to agonistic/non-agonistic functions on FXR and TGR5. * *p* < 0.05, ** *p* < 0.01, *** *p* < 0.001. Abbreviations: BA: bile acid, T: Taurine, CA: Cholic acid, CDCA: Chenodeoxycholic acid, DCA: Deoxycholic acid, MCA: Muricholic acid, UDCA: Ursodeoxycholic acid.

## Supplementary Materials

The following are available online, Figure S1: The major veins involved in bile acid reabsorption and circulation to the liver as well as sites of blood collection.
