# Supplementary figures and images for: Hepatic Bile Acid Reuptake in the Rat Depends on Bile Acid Conjugation but Not on Agonistic Properties towards FXR and TGR5
Source: Molecules. 2020 May 20;25(10):2371. doi: 10.3390/molecules25102371 (PMC7288213; doi:10.3390/molecules25102371)

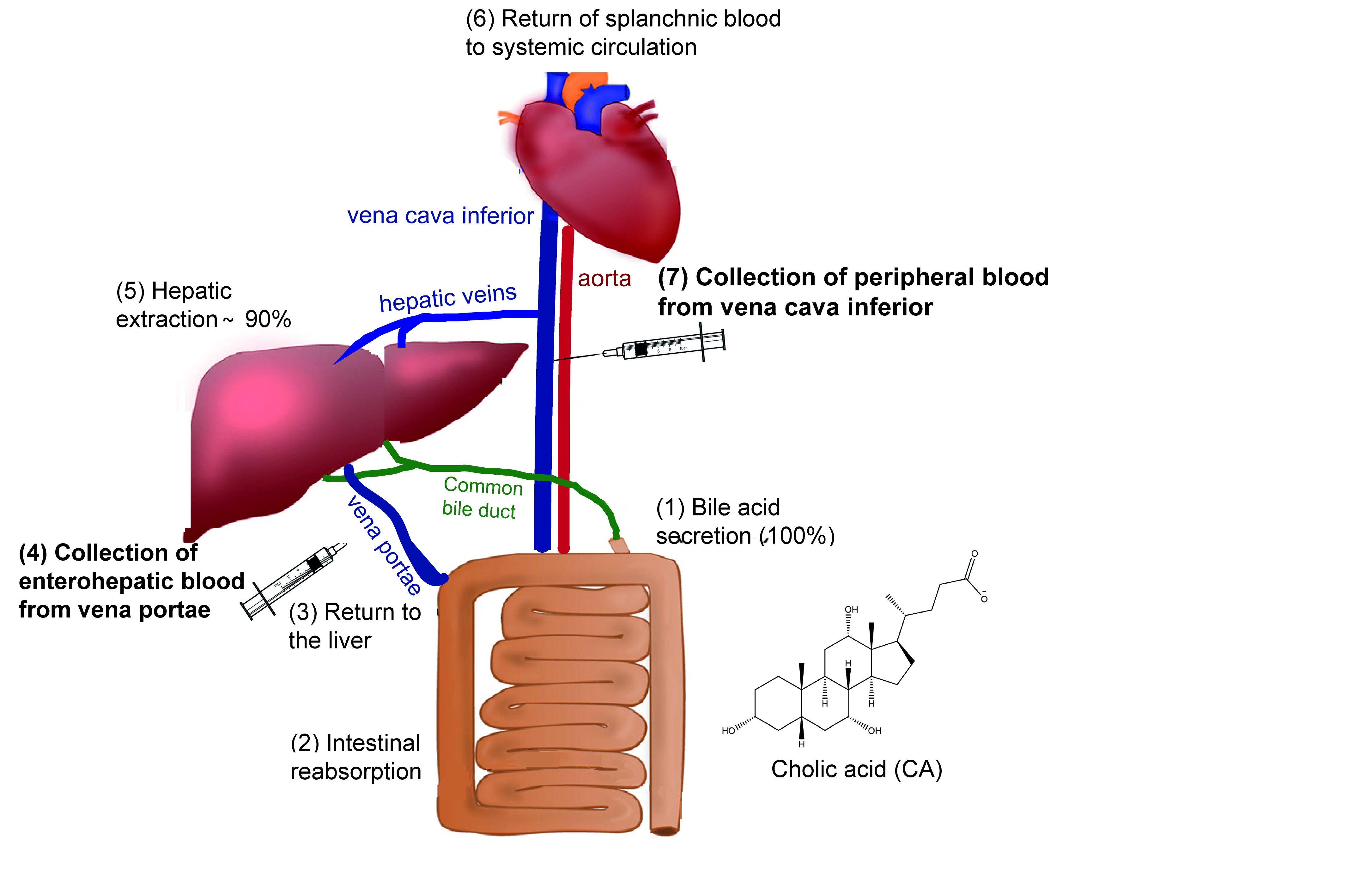

Supplement: Supplementary file 1 [file molecules-25-02371-s001.tif]
